# Supplementary material for: Patterns of Intron Gain and Loss in Fungi
Source: PLoS Biol. 2004 Nov 30;2(12):e422. doi: 10.1371/journal.pbio.0020422 (PMC532390; doi:10.1371/journal.pbio.0020422)
Supplement: Table S1 — Also available at http://genes.mit.edu/NielsenEtAl/. (4.3 MB ZIP). [file pbio.0020422.st001.zip › NielsenEtAl/html/156.html]

AN4226.1.NCU01595.1.MG03210.1.FG01446.1


```
 CLUSTAL W (1.82) Multiple Sequence Alignments - Introns Inserted


Sequence 1: AN4226.1	450 aa
Sequence 2: MG03210.1	408 aa
Sequence 3: FG01446.1	456 aa
Sequence 4: NCU01595.1	449 aa
Alignment Length: 465 aa
Number Identitical Residues: 186 aa
Alignment Score (without introns) 10690


MG03210.1 	----------------------------------------------------MERMFAAP
NCU01595.1	MTLLQQIKALSRNISAQQAPGNDVTRAPRNLAPELHPFERAREYKRALNAVKLERVFAKP
FG01446.1 	----MKIKALTRSASTQQVAGSDVTKQPRNLDSALHPFERAREYQRALNAVKLERMHAQP
AN4226.1  	-MPEQKIKALSRSTASQQAPGSAVARQPRNLDPAQHPFERAREYTRALNAVKLERLFAAP
          	     . .: : . ::.. ...  :  . .  .   . . : .   : .: .:**:.* *

MG03210.1 	FIAQLGKGHVDSVFSMTVDPESLSRVASGSADGVVKIWDLTSREEM0WHTTAHENIVKGM
NCU01595.1	FLGQLGNGHVQGVYSMAKDKNSLHTIASGSGDGIVKVWDLTTRDEA~WKAAAHNNIVKGM
FG01446.1 	FIGQLGNGHVDGVYSMAKDPNSLDHVASGAGDGIVKVWNLADRDEI~WHASAHENIVKGI
AN4226.1  	FLGQMGEGHVDGVYTMAKDPGSLERFASGSGDGVVKVWDLVTQGEV~WNNTAHENIVKGV
          	*:.*:*:***:.*::*: *  **  .***:.**:**:*:*. : *  *: :**:*****:

MG03210.1 	CFTKDQKLLTCASDRTIKLFSP-DQASDSAPLSTWLGNNAFTSLSHHRSKNSFAAASSV-
NCU01595.1	TFTNDKKLLTCATDG-IKLWDPYTKTDSPTPLATWQEGGPYTALSFHRNTNSFVASSGAG
FG01446.1 	TWTRDQKLLTCAADKTVKLFDPYNTPSNAAPISSWLGSGAFTSLSHHRSNNSFAAASSA-
AN4226.1  	CWTPDRKLLSCAADKTIKLFDPYNSSSESPPLATYLGQGAFTSLTHHRDQPFFAASSSQ-
          	 :* *:***:**:* ::**:.*   .....*::::   ..:*:*:.**.   *.*:*.  

MG03210.1 	-ISIYDLERHSAAP-EVLRWPTSTDTINSVAFNQVEQSILGSCASDRGIIL2YDLRTSMP
NCU01595.1	CIRIWDLE-HSTAP-QTIQWPNHSDTITDVCFNQVETSIVGSVGTDRSVIL~FDLRTNMP
FG01446.1 	-IHIYDLERHTAAP-EVLKWPTSVDTITDVAFNQVETSILGSCSNDRSIVI~YDLRTSTP
AN4226.1  	-ISIYDLSRPSSTPSQTLHWPTSVDTITSVAFNQTETSVLASTGIDRSIIL~YDLRTSSP
          	 * *:**.  :::*::.::**.  ***..*.***.* *::.* . **.::: :****. *

MG03210.1 	LHKTVLNFTCNKFAWNPMEAFNFAVASEDHNIYL2FDMRKIDRALNVLKGHVAAVMDVQF
NCU01595.1	VIKTVLKFAANRIVFNPMEAMNMAVASEDHNVYL~FDARNFTKAQNIQKGHVAAVMDVEF
FG01446.1 	VTKTVLKFASNRLSWSPMEAFNLAVANEDHQIYL~FDMRKMDRALNILKDHVAAVMDVEW
AN4226.1  	LSKLVLKLASNAVSWNPMEAFNFAVANEDHNVYM~FDMRKMNRALNVLKDHVAAVMDVDF
          	: * **:::.* . :.****:*:***.***::*: ** *:: :* *: *.********::

MG03210.1 	SPNGEELVSG1SYDRTIRLWKRDKGHSADIYHT1KRMQR~VFASAWTPDNKYVLSGSDD~
NCU01595.1	SPTGEELVTG~SYDRTIRVWRRDQGMSRDVYHT~KRMQR~VFRTTWTMDSKYLLSGSDD0
FG01446.1 	SPTGEELVSA~SWDRTVRLWNRDSGHSRDIYHT~KRMQR~VTAARWTPDARYILSGSDD~
AN4226.1  	SPTGEELVTA~SYDRTIRLWNRATGHSRDIYHT~QRMQR2VFSAKFTPDNKYVLSGSDD~
          	**.*****:. *:***:*:*.*  * * *:*** :**** *  : :* * :*:****** 

MG03210.1 	GNVRLWRARASQREGVKSARQRQAEEYNQAVTARYAHMPEIRRIKNHRHVPKVIKKAGEI
NCU01595.1	GNVRLWRANASERSGVKATKQRQALEYNKTLVERYSHMPEIKRISRHRHLPKVVKKAAEI
FG01446.1 	GNVRLWRANASRREGVKSARQRQALEYNDALIERYQHMPEVRRIHRHRHVPKVLKKAGQI
AN4226.1  	GNIRLWRANASDRSGIKSARQRTKLEYDQALVQRYAHMPEIKRIKRQRHVPRTIKKAREI
          	**:*****.** *.*:*:::**   **:.::  ** ****::** .:**:*:.:*** :*

MG03210.1 	KSEELRAIKRREENERKHSSK-KFEKRKA~EREKMILAKEK2KSKKILRLMFPR
NCU01595.1	KREELAAIKRREENERKHSVK-QFEKRKA~EREKAILAKLE~------------
FG01446.1 	KSEELKSIKRREENERRHSKK-QFERRRG1LQKAWKGIAWR~SGRRLMGNLDD-
AN4226.1  	KNEELAAIKRREENIRKHAKKSTLRARQS~EREKMILAQEK~------------
          	* *** :******* *:*: *: :. *:.  ::       .
```
